# New Modes of Practice: A Framework Analysis of School Nurses' Accounts of Working With Children and Young People During COVID‐19

**DOI:** 10.1111/jan.16490

**Published:** 2024-10-01

**Authors:** Sarah Bekaert, Maisie Rawlings, Dominic Shariff, Dana Sammut, Georgia Cook

**Affiliations:** ^1^ Faculty of Health and Life Sciences Oxford Brookes University Oxford UK; ^2^ Centre for Healthcare and Communities University of Coventry Coventry UK

## Abstract

**Aims:**

To identify new and accelerated modes of practice used by school nurses during the COVID pandemic. To create a quick reference infographic bringing together experiential evidence on the range and considerations regarding different modes of practice for use by the school nursing community of practice.

**Design:**

A descriptive qualitative secondary data analysis of open‐ended questions in a survey, and focus groups with school nurses. The pragmatic aim was to focus on changes in school nurse modes of practice to ensure continued engagement with children and young people, and school nurses' experience of the benefits and challenges of these modes of practice.

**Methods:**

Data were collected from 98 school nurse participants across a United Kingdom‐wide survey (n78) in April to May 2022, and focus groups (n20) in June to July 2022, within the School Nursing in the Time of COVID project. Data from the seven open‐ended questions in the survey and four questions from the focus group were analysed using the framework approach.

**Results:**

Modes of practice fell into two categories: individual assessment and support (video‐calling platforms, telephone contact, virtual messaging, walk‐and‐talks and home visits) and group support (wellbeing approaches, social media). Considerations for these modes rooted in school nurses' experience are described. Interpretations were used to create the summarising evidence‐based infographic as a quick reference resource for school nurses.

**Conclusion:**

There was no ‘one size fits all’ approach. The modes used by school nurses were adopted or developed out of necessity or resource availability and in response to the specific needs of individuals or groups. The developed infographic provides a quick reference guide to deliver the expressed need for knowledge exchange within the school nursing community by participants in the original studies and can be used to inform current school nurse practice.

**Implications for the Profession and/or Patient Care:**

The developed evidence‐based infographic has stand‐alone value. It has the potential to raise awareness of the range of different strategies that can be used to facilitate and/or enhance engagement with children and young people, equip school nurses with knowledge to foster innovative and responsive practice and aid critical reflection in a complex post‐pandemic landscape. The infographic is a unique resource and is a first step in knowledge exchange based on experiential learning. The resource will be used as the foundation for future work to develop a co‐created training resource for school nurse students (undertaking the Specialist Community Public Health Nurses course) and/or continuing professional development resource for established school nurses.

**Reporting Method:**

This study has been conducted and reported in accordance with COREQ guidelines for qualitative research.

**Patient or Public Contribution:**

A consultation group was closely involved with the planning, conduct and analysis of the original studies. This group consisted of representatives from professional organisations SAPHNA (School and Public Health Nurses Association) and the CPHVA (Community Practitioners' and Health Visitors' Association), a school nurse and a member of the public. SAPHNA have continued in their consultative role for this study and has provided content and usability feedback regarding the infographic. Early findings have been presented to the SN community of practice, and feedback invited, through presentation separately at the CPHVA and SAPHNA annual conference.

**Summary**

- What is already known?
  - In the United Kingdom, school nurses are registered public health nurses who work with children aged 5–19 according to the Healthy Child Programme. The Healthy Child Programme operates under a universal and progressive system, outlining a schedule of interventions for all children, as well as those who need additional support and input.
  - Whilst adaptations to practice were manifold during pandemic restrictions, school nurses' work with children and young people saw a universal expansion of digital communication methods, such as video‐calling platforms, telephone and text messaging services, which were utilised for both online consultations and interventions as well as for staying in contact more informally.
  - Challenges accompanied these changes in practice, most notably a loss in school nurses' perceived quality of contact with children and young people, largely attributed to a reliance on virtual contact and the increased demands on school nurses' time.
- Implications for practice/policy
  - This analysis is at the forefront of addressing the gap in evidence in demonstrating the clinical effectiveness of the range of modes adopted to facilitate SN practice with children and young people during the pandemic to inform potential post‐pandemic use.
  - The findings provide practitioners' experiential evidence regarding the use of a range of modes of practice introduced or accelerated during the pandemic, and often in continued use. This facilitates shared experience and learning for the school nurse community and enhances practice.
  - The next stage is to develop a co‐created training resource for school nurse students and continuing professional development for registered school nurses from this evidence base. The one‐page infographic will be used to stimulate guided reflection on different mode's usefulness and feasibility in practice.
- What this paper adds and contribute to the wider global clinical community?
  - This study is a novel contribution to the growing school nurse‐specific evidence‐base regarding modes of practice with children and young people. Findings come from and inform the school nurse community of practice.
  - This is the first UK study/resource to provide details on the range of new and accelerated modes of practice utilised by SN during the pandemic with the aim of supporting post‐pandemic practice.
  - The resulting quick‐reference evidence‐based infographic is a unique summary of the new and accelerated modes of practice and associated considerations which facilitate knowledge exchange within the SN community of practice.

## 1 Introduction

The COVID‐19 pandemic led to remote school curriculum delivery in many countries across the world for significant periods of time (Munoz‐Najar et al. 2021; Thorn and Vincent‐Lancrin 2022; Cook et al. 2022). The United Kingdom saw a series of lockdowns and phased school re‐openings. The first nationwide lockdown spanned from March to June 2020, followed by a gradual return in social contact alongside additional protective measures such as small social ‘bubbles’, maintaining social distancing, and wearing face masks. Further local lockdown regulations commenced in October 2020, lasting until early March 2021, with schools reopening under local tiered restrictions that continued until mid‐July 2021 (Institute of Government 2022). School closures meant that in‐person schooling ceased and moved online for all except vulnerable children, such as those on the child protection register, and the children of key workers (mainly National Health Service [NHS] and education staff) (Sammut et al. 2022).

In this dramatically changed and inconsistent period, school health services, largely delivered in‐person on school premises before the pandemic, had to rapidly adapt their approach to ensure that children and young people (CYP) continued to receive health promotion, intervention and safeguarding support. In the UK school nurses (SN) are registered public health nurses who work with children aged 5–19 according to the Healthy Child Programme (HCP) (Office for Health Improvement and Disparities 2016). The HCP operates under a universal and progressive system, outlining a schedule of interventions for all children, as well as those who need additional support and input. Further, Public Health England (2021) and the School and Public Health Nurses Association (SAPHNA) (2021) set out the role of the SN in three areas: universal, personalised or targeted, and specialist, with safeguarding forming a key component across all three. In the United Kingdom, the school nursing service is predominantly commissioned by Local Authorities (LA) and a few National Health Service (NHS) providers. As such, SNs work within schools but are not employed by schools. The exceptions are fee‐paying schools that directly employ a school health team. However, LA or NHS providers are responsible for certain universal services to CYP attending fee‐paying schools, that is, immunisations or the National Child Measurement Programme. SNs are Specialist Community Public Health Nurses (SCPHNs), a separate registration on the Nursing and Midwifery Council register, and require a postgraduate qualification which focuses on child health, health promotion, public health, and education. Commensurate with the focus on school nursing, ‘children and young people’ in this paper refers to all school‐aged children (5–19 years) for whom SNs have responsibility, as defined by the commissioning guide for the HCP (Public Health England; PHE 2021).

## 2 Background

An international scoping review of the literature, conducted as part of a wider project by the authors, identified how SNs across the globe adapted their practice when faced with pandemic‐related challenges. This review highlighted how SNs continued their provision of services with CYP and families, alongside their ongoing work with the wider multidisciplinary team, and their work as a school nursing team (Cook et al. 2022). Whilst adaptations were manifold, SNs' work with CYP saw a universal expansion of digital communication methods, such as video‐calling platforms, telephone, and text messaging services, which were utilised for both online consultations and interventions as well as for staying in contact more informally (e.g., Combe 2020; Lee et al. 2021). General health education was also frequently delivered via digital platforms (e.g., Martinsson, Garmy, and Einberg 2021). Alternative environments, such as children's centres, empty offices, and outdoor interactions, were explored for face‐to‐face service delivery when permitted (e.g., Barbee‐Lee et al. 2021). However, challenges accompanied these changes in practice, most notably a loss in SNs' perceived quality of contact with CYP, largely attributed to a reliance on virtual contact and the increased demands on SNs' time (Martinsson, Garmy, and Einberg 2021).

In order to capture a UK‐specific perspective consequent to the scoping review; where evidence was predominantly US‐centric, and the need to identify new and accelerated modes of practice used by SN during the COVID‐19 pandemic, and the benefits and challenges of these; our original study, School Nursing in the Time of COVID, explored the experiences of SNs in the United Kingdom through a survey and interviews/focus groups (Sammut et al. 2022; Bekaert et al. 2023). SN participants, particularly within the interview/focus group study, reported advantages associated with their involvement in this research. Most notable among these was an appreciation of the opportunity to learn about the different practice modes used by other SNs nationally, including discussions about the associated benefits and challenges of these. Participation also encouraged professional reflection, with many SN participants expressing intentions to implement changes in their local practices based on newfound insights. It was also suggested that a resource that brings this information together in an accessible format would be valuable for continued learning and reflection. Therefore, the pragmatic aim of this study was to provide information rooted in real‐world experience with transferable potential to the United Kingdom, and likely global, SN community. Subsequent to the pandemic, many of these modes have been continued or discontinued without review of their feasibility and usefulness to practising SNs. In response to the voiced need of the SNs in the focus groups, the broad aim of this study was to develop a quick reference infographic to facilitate knowledge exchange within the UK SN community and inform current practice. This would be a simple resource that would bring together experiential evidence, raise awareness, and encourage reflection on the range and use of different modes in current and future practice. The research questions for this study were: (1) what were the changes in SN modes of practice to ensure continued engagement with children and young people and SN during COVID restrictions and (2) what were SNs' experience of the benefits and challenges of these modes of practice?

## 3 The Study

The first stage of this study was to re‐analyse the original qualitative data (from Sammut et al. 2022 and Bekaert et al. 2023) with the explicit intention of identifying: (1) the new and/or accelerated modes of practice described by SNs to ensure continued engagement with CYP during the pandemic and (2) the reported benefits and challenges associated with use of these practice modes during the pandemic. This study was conducted and is reported in accordance with COREQ guidelines for qualitative research (Tong, Sainsbury, and Craig 2007).

## 4 Method

A descriptive qualitative secondary data analysis of open‐ended questions in a survey and focus groups with SNs was undertaken. It is recognised that open‐ended survey responses are limited; the aim of examining both the survey and focus group data leads to ‘volume’ of findings from the survey data and exploration of these findings in the focus group data, hence grouping the data for analysis. We have not sought to quantify any findings and therefore the findings are presented as narrative description. Secondary analysis was conducted on data from 98 participants across two studies. A convenience sampling approach was employed to reach UK‐based school nurses in both cases. Participants were recruited to the original studies (Sammut et al. 2022; Bekaert et al. 2023) through adverts disseminated via professional SN organisations, including social media and the research teams' professional networks and social media.

Study 1 was a UK‐wide survey conducted between April and May 2022. The anonymous embedded link in the advert led to an online Qualtrics‐hosted survey. The landing page included a brief description of the study, a participant information sheet and the ability to provide consent by checking a box. In order to maintain respondents' anonymity, the survey did not capture or record Internet Protocol addresses and only regional geodata. Participants could exit the survey at any time, with only fully completed and submitted surveys included in the analysis (*n* = 78).

Respondents to the survey were invited to participate in follow‐up focus groups. Focus group participants were also recruited via the same social media channels as for the survey. The focus groups were conducted between June and July 2022. Due to pandemic restrictions, focus groups were conducted online. To support facilitation in an online environment, each session had a maximum of four participants. Sessions were run by facilitators experienced in focus group/interview methods, and supported by co‐facilitators with SN expertise. Where only one participant signed up to, or joined a session, they were offered the opportunity to continue as a one‐to‐one interview, or reschedule to an alternative session. Where participants opted to proceed one‐to‐one, co‐facilitators exited to avoid outnumbering the interviewee. Sessions were audio recorded via Zoom, with automatic transcription settings enabled, with recorded transcription checked for accuracy against the audio‐recording. Twenty SNs participated, with sessions lasting between 50 and 80 min. It is possible that some participants may have participated in both the survey and focus groups. However, as survey responses were anonymous, any potential overlap was not identifiable.

For the current analysis, data from the seven open‐ended questions in the survey and four questions from the focus groups were analysed (see Box 1 for details of the questions). This study received Oxford Brookes University Research Ethics Committee approval (registration no. 211550).

BOX 1 **Qualitative questions in the survey and questions asked in the focus groups/interviews.**

**Qualitative Survey Questions**

Can you briefly explain changes in children's, young people's or families' contact with the school nursing service during COVID‐19?

Please can you provide a summary of the feedback received or evaluation conducted on the school nursing services offered?

Did you experience any particular challenges or barriers to perform your school nurse role?

Can you give a brief description of how COVID‐19 restrictions impacted your ability to work with vulnerable children, young people and families that were already known to you?

Can you give some examples of where school nursing partnership working has improved?

Can you give some examples of where school nursing partnership working has been harder or more challenging?

Do you have anything else to add about your experience of being a school nurse during the pandemic and its impact on your practice?

**Focus group/interview questions**

Can you tell me about the different modes of delivery used with children, young people and families that evolved as a result of COVID‐19 restrictions?

How did the COVID‐19 restrictions affect your ability to support vulnerable children, young people and their families, Children in Need and those on the child protection register?

School Nurses work with a range of professionals (e.g., with education, social care, community health services, emergency departments, sexual health services, child and adolescent mental health services, community children's nursing teams, police services and substance misuse services)—can you describe the impact of COVID‐19 on these partnerships?

Do you think you will carry on beyond the pandemic with any new ways of working with children, families and professionals that accelerated or emerged during lockdown and restricted access?

Two pre‐registration nursing masters (MSc) students (MR, DSh) collaboratively analysed the data. This was undertaken manually through a shared, password protected, Google drive folder. Data were anonymised prior to analysis. MR and DSh maintained weekly contact with the wider research team (SB, GC and DS) to discuss the analysis. The wider team included three researchers from the original research team who were involved in primary data collection: two post‐doctoral, and one pre‐doctoral. Two were also registered nurses—one Adult and one Children's Nurse—and the third had a PhD in psychology.

Rigour has been examined through Lincoln and Guba's (1985) framework for establishing the rigour or trustworthiness of a qualitative study. Credibility, or the truth of the findings, is maintained through closer examination of specific questions asked in the original studies. These questions focused on changes in modes of practice and the benefits and challenges of these changes in practice. Examination of the two datasets gives ‘volume’ of information through the survey, which is then explored in more depth in the focus group data. In addition findings were reviewed and affirmed by a consultation group that represented and included SNs which affirms real‐world application. Survey and focus group data were gathered from across the United Kingdom, and represents the experiences of SNs in state and independent, primary and secondary schools, with full and part‐time contracts. Staff SNs, Specialist Community Public Health Nurses in Schools, and SN team managers were represented. Therefore, the experience of SNs across the United Kingdom is represented and the findings are likely to be transferable. Dependability was sought through protocol, findings and recommendations review by the study consultation group which consisted of representatives from a school nursing professional organisation and an experienced practising SN. Confirmability was achieved through systematically applying Gale et al.'s (2013) Framework Approach to the dataset as a systematic, and therefore replicable, way to manage and analyse a large dataset with predetermined criteria, and regular review and consensus discussion by the research team.

### 4.1 Analysis

This secondary analysis adopted a pragmatic approach. Pragmatism grounds inquiry in the lived experience of individuals, identifying useful knowledge which can be utilised in effecting change (Allemang, Sitter, and Dimitropoulos 2022; Kelly and Cordeiro 2020). The original two qualitative datasets (from 98 participants) were re‐examined to identify any data relating to the questions: What are the changes in modes of practice described by SNs to ensure continued engagement with CYP? How do SNs describe the benefits and challenges of these modes of practice? The goal was to extract the practical learning of SNs during the pandemic that could be useful for future SN practice and form the basis for the information resource. The qualitative data were re‐examined drawing on a framework approach (Gale et al. 2013). This approach was suited to the aim of the study to bring experiential evidence to the SN community of practical tools to enhance practice rather than seek deep qualitative findings and new meanings. As such Gale's framework approach was ideal as a systematic way to manage and analyse a large dataset with predetermined criteria and to organise and categorise the collective information with transparency and rigour (Gale et al. 2013; Ward et al. 2013; Goldsmith 2021).

The seven stages of the framework approach were adapted for this analysis: (1) transcription (previously undertaken for the primary study); (2) familiarisation with the data; (3) coding; (4) developing a working analytical framework (informed by the research question); (5) applying the analytical framework; (6) charting data into the framework matrix; (7) interpreting the data (Gale et al. 2013). In this study, these processes were broadly divided into two phases: phase 1, which included stages 1–6 of the framework approach for the complete dataset; and phase 2, which included stage 7, interpreting the data, which was then used to create an evidence‐based infographic to summarise findings. The research team then collaboratively discussed and developed various iterations of a visual infographic which encapsulated the research study findings. Key stakeholders from the original study (SN, professional body representatives) reviewed and provided feedback on the infographic. Further feedback was gathered from the SN community of practice at two professional conferences.

#### 4.1.1 Phase 1—Framework Analysis

As transcription (stage 1) had already been undertaken by the original research team (SB, GC, DS), the first stage of analysis for this study involved the two MSc students (MR, DSh) reading through the survey and interview/focus group data for familiarisation purposes (stage 2). A matrix was prepared by SB, GC and DS to capture data relating to the specific questions being asked of the data that detailed: the change in practice modes identified (which were treated as the ‘codes’) and quotes that evidenced the change in practice and any associated benefits and/or challenges. As the a priori aim of this study was to identify changes in practice, stage 4, developing a working analytical framework, preceded coding (stage 3). MR and DSh then coded according to this analytic framework (stage 5). Changes in modes of practice (new and accelerated) were identified in relation to SNs' work with three distinct groups: SN teams; CYP and families and the wider multidisciplinary team. It was important to identify who (and how) these different groups were affected by practice changes, as some were more, or less, beneficial depending on which group they affected. For example, online meetings were considered to benefit professional communication more than SN‐child interactions.

The matrix also had a column for researcher thoughts or comments, or analytic memos, on the particular codes to provide a reflective space for the researchers during the analysis process. Please see Table 1 below for an extract from the first stage data extraction matrix.

**TABLE 1:** An extract from the first stage data extraction matrix.

	Change in practice (new, accelerated, increased)	Benefits	Challenges	Notes
SN team				
Redeployment	Q 12.2 Staff taken off role to deliver COVID vaccinations		Q12.2 Many staff redeployed, role became safeguarding and immunisation	
	Q12.2 We lost half our workforce due to redeployment	—	Q16 the few left had to manage everything	SN service seen as dispensable
			Q16 Those left were overwhelmed by safeguarding	SCPHN students also redeployed

During this charting stage (6) of the analysis, the quotes extracted were broad, with any change in SN practice noted. This included changes that were out of the control of SNs, for example, redeployment. In the interpretation stage (7) the matrix was refined to include only codes representing practices that SNs had an active role in changing. This aligned with the pragmatic aim of the study to identify experiential evidence which could be directly beneficial to SN practice and implementable at a practitioner level. MR and DSh met regularly with SB to discuss queries and reach consensus regarding coding, organisation of data and analysis.

#### 4.1.2 Phase 2—Infographic Development

At the final interpretation stage (7) In order to focus on SNs' work with CYP specifically, subsequent analysis focused on this column of the matrix only. SNs' engagement with families were only included when impossible to separate from the context of SNs' work with CYP; for example, during conversations that included CYP and families together, as opposed to communication that exclusively involved family members. Interpreting the data entailed two further steps. First, grouping codes by similarity. For example, Zoom, Teams, Google Meet, etc. were grouped under ‘video calling platforms’. The benefits and challenges identified were then combined into a ‘general considerations’ for each code and supporting illustrative quote. See Table 2 for an extract from the matrix developed at this stage.

**TABLE 2:** An extract of grouped codes.

Generic change	Considerations	Illustrative quote
All video based platforms (video calling services, generic virtual)	Benefits	FG6 (P10)—You can sometimes see a very different dynamic between family members, when they're in their own home, as when they're, you know, sat in front of you in a school or a clinic… and you can also see home conditions
	Increased accessibility for parents Can increase CYP attendance Increased flexibility (timing, location), wider reach of the school health service offer Increased accessibility of the SN Gain a wider picture/sense of the child's home context	Q18.2 (P74)—Therefore it wasn't always possible to speak privately with the child due to other's being present in their home. This may have meant they were less likely to make disclosures
	Challenges	FG3 (P6)—What worries me is about issues around digital poverty, not all of our children and families have got access to mobile phones or laptops
	Safeguarding concerns (child may not be alone/able to speak freely)	

The codes were then organised to represent two categories: changes in modes of practice relating to working with individual CYP, and those that facilitated practice with CYP as a population. The individual‐related codes were video calling platforms, telephone contact, virtual messaging, walk‐and‐talks, home visits groups/codes. The population‐related codes were well‐being approaches and social media groups/codes. Please see Table 3 for the final categories, and codes within each category identified.

**TABLE 3:** Final organisation of categories identified.

Category	Category
Individual assessment/support	Population support
Codes	Codes
Video consultations Telephone Virtual messaging Walk‐and‐talk Home visits	Well‐being approaches Social media

The infographic was then developed based on this categorisation with the SN community in mind ‐ as a quick reference, visually appealing resource.

## 5 Results

### 5.1 Characteristics of the Sample

The survey data represented SNs across state (88.6%), independent, mainstream and special education needs schools working in both primary (89.9%) and secondary settings (92.4%). Participants were based in England (*n* = 69), Scotland (*n* = 6), Wales (*n* = 1), Northern Ireland (*n* = 1), with one unidentifiable postcode. The majority (78.5%) held the SCPHN qualification. Participants reported they had been working as SNs for a mean of 12.35 years (ranging from 1 to 39 years).

The focus group/interview data represented 20 school nurses, based in England (*n* = 18) and Scotland (*n* = 2). Due to the smaller number of participants and the need to protect anonymity demographic details were not routinely recorded but were evident within the conversations. Participants ranged in role and experience and included team or clinical leads, advisors and managers, and practice educators. Participants also worked across state and independent schools and with both primary and secondary school‐aged children.

### 5.2 Groups/Codes

**FIGURE 1:**
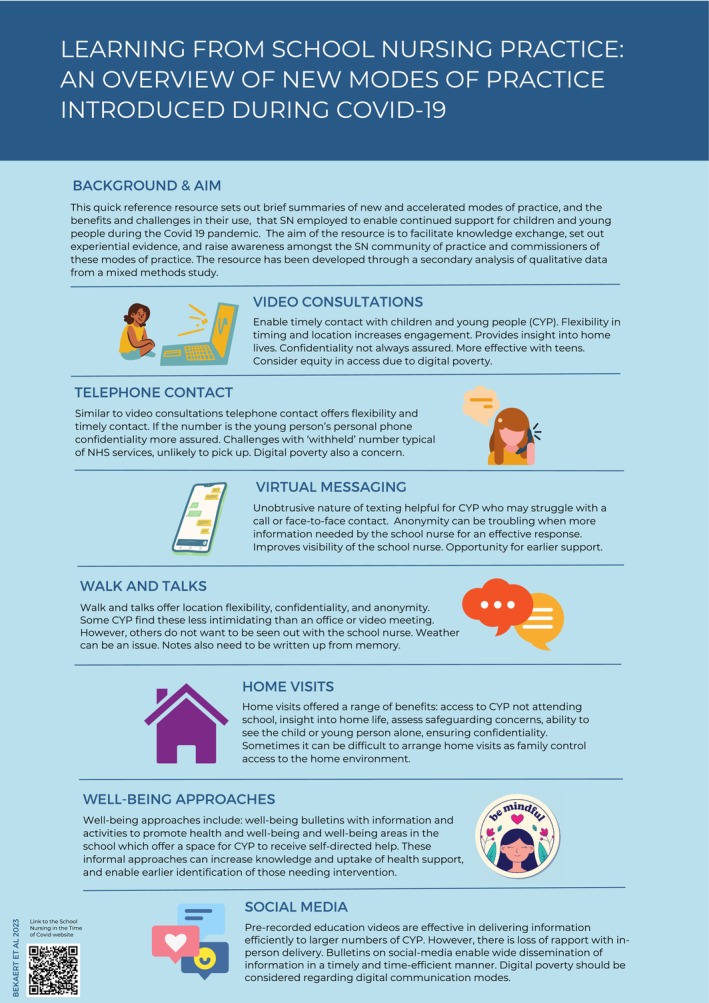
Learning from school nursing practice: An overview of new modes of practice introduced during COVID‐19.

The groups/codes are presented alongside practitioner‐reported benefits and challenges, and illustrative quotes. This section represents the detailed evidence underpinning the resource. Quotes are presented alongside an anonymised reference for the location of the quote and participant number, that is, focus group/participant (FG/P1), survey question/participant (Q/P6). This section concludes with the quick‐reference infographic resource (Figure 1).

### 5.3 Category: Individual Assessment and Support

#### 5.3.1 Code: Video‐Calling Platforms

There was widespread use of video‐calling platforms during the pandemic as these allowed SNs to contact CYP without the need for physical proximity.

**Benefits—**Video‐calling platforms were generally praised for their ability to allow continued engagement between SNs and CYP without the need to meet face‐to‐face. There were also other benefits to video‐calling, such as the insight that the SNs felt they gained into the CYP's home life and environment that they would not have access to in school:

‘You can sometimes see a very different dynamic between family members, when they're in their own home, as when they're, you know, sat in front of you in a school or a clinic… and you can also see home conditions as well, some elements of that, you know, which gives you that context doesn't it. It's the life that that young person is living’ (FG/P10).

**Drawbacks**—The most frequently reported concern with video‐calling platforms was the difficulty in building rapport with CYP through this medium. SNs felt that screens created a barrier to effective communication and relationship‐building. This was particularly noted for meetings that involved difficult conversations or emotional content. SNs were not happy with the abrupt manner in which such meetings ended, and the inability to offer informal support. This was particularly noted for child protection core group meetings (where key professionals meet with the family to review a child protection plan), which were also conducted online, where SNs spoke about their inability to comfort family members as effectively as they would have liked, or to provide follow‐up support. One SN manager commented:

‘School nurses were saying they were attending child protection conferences via Teams and parents would just leave. And…you can't follow them out and have a conversation with them, calm it down. You don't know what's going on, you don't know whether there's something else going on in the house, they just leave’ (FG/P5).

#### 5.3.2 Code: Telephone Contact

The use of telephones for appointments and the provision of wider support to CYP was reported to have significantly increased.

**Benefits—**Much like video calling platforms, there were many benefits stated to the use of telephone calls that centred around flexibility and accessibility: ‘They'll talk to you on the phone so if we can be as flexible as possible, we'll be able to reach out to a larger population of our young people’ (FG/P18). SNs also reported that, generally, young people were happy with telephone contact: ‘There's a service called Accurx which the school nurses use, where you can arrange video or telephone consultations…the feedback is that young people prefer it… it's more convenient, is what we've had back’ (FG/P12).

**Drawbacks**—SNs were mindful of the tension between maintaining contact with vulnerable CYP, and the possible limitations in confidentiality when speaking to a young person on the phone in their home environment:

‘I think the phone calls that I did, you know I did manage to get hold of a few of them, but I just felt that they, the kids, were at home and parents were there and they might be listening… Whereas when you're in school, you know it's a safe environment, they're away from the house. That was the worrying thing really with these vulnerable kids, it's like they're not going to talk if they think mum or dad's listening’ (FG/P20).

#### 5.3.3 Code: Virtual Messaging

Virtual messaging included a variety of different communication approaches, from text and WhatsApp messages, to online general services like Discord (a public online messaging platform), and tailored communication platforms such as ChatHealth (a confidential text messaging service used in the NHS that enables users aged 11–19 to contact their local SN team for advice and support). SNs reported that some of these formats were already in use pre‐pandemic but increased during this time, while for others these methods were introduced during the pandemic.

**Benefits**—SNs reported the unobtrusive nature of texting helped with engaging with CYP who felt anxious about a call or face‐to‐face session. The anonymous nature of many texting services was perceived by SNs to encourage some CYP, who otherwise would have found it difficult to engage, to communicate with them. For example, SNs reported that for some, anonymity helped young people to feel like they did not have to explain themselves:

‘… doing a WhatsApp conversation has been a great introduction, rather than meeting face to face [with CYP] to talk about your sex life (for example) with somebody they've never ever met before. And they like that…’ (FG/P10).

Having a centrally managed virtual messaging service (i.e., ChatHealth) ensured that young people could consistently access advice and support, and also avoided reliance on individual practitioners: ‘We do encourage ChatHealth as a point of contact, so we try not to give our phone number to totally rely on because, obviously, there are times when we are not available or leave etc.’ (FG/P11).

**Drawbacks**—Some SNs reflected that the anonymity that these platforms afforded could present difficulties if they felt they needed more information in order to respond effectively: ‘Sometimes you could get the feeling that it wasn't working this virtual environment, you could do texting and things like that, and sometimes you just ended up really worried by one word in a text thinking what is going on for that young person’ (FG/P17).

SNs also reported that they were unable to convey tone of voice or body language, potentially closing another avenue for obtaining information: ‘The convenience, the increased attendance… from students (was beneficial), but then, at the same time it's hard to read body language, there's a lack of visual cues, and I think almost it's harder to build rapport really’ (FG/P8).

#### 5.3.4 A Note on Digital Poverty

For these three formats—video calling platforms, telephone contact and virtual messaging—SNs also voiced more general concerns around digital poverty, cognisant of the fact that some families did not have access to certain technologies, resulting in reduced access to health services: ‘What worries me is about issues around digital poverty, not all of our children and families have got access to mobile phones or laptops’ (FG/P6).

#### 5.3.5 Code: Walk‐and‐Talk

An alternative method taken up to deliver a typical face‐to‐face interaction was the ‘walk‐and‐talk’. A walk‐and‐talk is a session with a child or young person where they meet up with the SN and go for a walk for some or all of the duration of the interaction. This approach became popular during the pandemic as it enabled face‐to‐face contact during times of social contact restriction.

**Benefits—**Walk‐and‐talks were welcomed by SNs and reportedly some young people, due to the flexibility of options for meeting location and time, and the opportunity for in‐person contact: ‘I think the girl was probably a bit anxious of coming into my office at school, so I did a walk‐and‐talk after school and that worked well’ (FG/P9). SNs reported that many pupils opened up more in these sessions as they found it to be less intimidating than being sat face‐to‐face in an office or video call:

‘I found the walk‐and‐talks good and I think they were quite well received by young people as well, you know, you've not got that sort of… sometimes awkward sitting face to face with someone where you're like ‘oh where do I look?’ and you know, when you're walking side by side you've not got that intense eye contact, and I think conversations just flow a bit more naturally’ (FG/P9).

**Drawbacks—**Confidentiality with walk‐and‐talks is not guaranteed, as, depending on the location, a young person may be seen by others with the SN. SNs reported that not all young people welcomed this possibility: ‘They didn't want to be seen out with the school nurse, walking and talking, wherever we were’ (FG/P8). Some SNs also noted that unreliable weather could be a problem when organising such sessions: ‘Just had to take a potluck on what the weather was doing, you know if it was raining, well, you can make the appointment, you'd still go out, you know’ (FG/P9). In addition, SNs spoke about the challenge of being unable to write notes while walking, and remembering the detail of what was said:

‘And what I did find was that, when you're in an office, you can sort of take notes as you go along, but with a walk‐and‐talk, you were kind of having to mentally clock, like, ‘talked about hobbies, right I'm talking about sleep, right I'm talking about friends’ you know, and all those sorts of things that we naturally talk about, and you had to kind of do it all on memory. So, I would get back into the car and just tap away really on the iPad just to try and make the notes as timely as possible’ (FG/P9).

#### 5.3.6 Code: Home Visits

Home visits, much like walk‐and‐talks, were utilised prior to pandemic restrictions, however they were increasingly used by SNs during this time.

**Benefits**—Home visits also offered a mode of assessing safeguarding concerns, allowing SNs to see CYP who were known to social services, or about whom there were worries:

‘It became more and more important that we, somebody, had eyes on them basically, I suppose, and that's when we would go and do the home visits. Especially if they were, say, looked after children, they weren't attending and looked after at home, which is an even higher risk isn't it ‐ they weren't attending, they weren't seeing…nobody had eyes on (the child)…’ (FG/P10).

Home visiting was said to provide unique insights into a child or young person's home life and their family dynamics: ‘… making a better connection with the family and seeing context, you know I think it's one thing to discuss it but to actually see it, and so that helped and you know the ones that were willing to do home visits’ (FG/P19).

An additional reported advantage of home visiting was that it provided access to CYP that were not attending school as restrictions were lifted:

‘Historically it's like well I can't see them because they're not in school, but now I think because we've had that (home‐visits) because we've done it with Covid and we're kind of more used to it …’ (FG/P20).

Due to the benefits afforded by home visits some SNs voiced that they would be carrying on this practice post‐pandemic:

‘I think next year I will try and you know, try and reach some of those really vulnerable kids who aren't coming into school and do that, possibly do like a little walk‐and‐talk with them or home visit’ (FG/P20).

**Drawbacks**—SNs noted that this type of access was controlled by families who were within their rights to revoke access at any time, and this was particularly concerning in those instances where safeguarding concerns were present: ‘It was harder to reach the vulnerable children that were under social services plans as families did not always consent to home visits’ (Q/P51).

### 5.4 Category: Population Support

#### 5.4.1 **Code:** Strategies Focused on CYP Wellbeing

Several SNs reported leading or contributing to new approaches to support children and young people's well‐being more generally. These included newsletters or bulletins distributed in or by school, and the set‐up and running of well‐being areas in schools. Bulletins were reported to include advice to support mental well‐being such as grounding and mindfulness techniques, and contained practical elements such as mindful colouring. Well‐being areas provided quiet, safe areas, with optional activities such as ‘make and do tasks’ for CYP to come to when they needed time and space away from the school timetable or during scheduled breaks. These areas were supervised by a school staff member and/or the SN.

**Benefits**—SNs found that well‐being bulletins were well‐received by the school population:

‘With our marketing team, we started putting together a well‐being bulletin. So, I would look at you know, whether it was like mindful colouring, mindful podcasts, you know gathering and sharing that information. So, we started it initially, doing that for pupils, and then we started doing it for staff’ (FG/P4).

Well‐being areas were noted to have several advantages for SN practice and CYP's health: for example, providing a space for CYP to seek help in an informal manner. These areas also allowed SNs to identify CYP who might need formal intervention at an earlier stage, to ‘keep an eye on’ the general school population, and deliver support to those without formalised support plans:

‘We set up a well‐being hub in a different part of the school, with sofas, cushions, plants, colouring in books and things, and that was staffed by guidance and ourselves and other teachers that wanted to help, with the understanding that it was a nice chillout zone for pupils to use at break and lunch time, if they were struggling to integrate back into school and things. And that was used really well… (we were able to identify) one or two that hadn't come across our paths before, that hadn't said to anyone that they were struggling…’ (FG/P1).

No disadvantages were voiced for these modes.

#### 5.4.2 Code: Social Media

Pre‐recorded videos and social media platforms were cited as options through which SNs could communicate health promotion and educational messages to CYP as a population.

**Benefits**—these modes were noted as having benefits for increasing reach and efficiency with SN time. For example, pre‐recorded videos were widely used as a rapid way of delivering important public health and health promotion information to a large number of CYP:

‘We videoed the presentations… and sent them out to schools virtually. This meant we were able to reach a large number of hard to reach young people. Schools gave positive feedback about this piece of work’ (Q/P34).

The use of social media also allowed for the wider dissemination of information and education from a central location. In addition to the efficiency benefits, participants indicated that CYP knew where they could find information about their local service, and were able to access this easily and receive regular updates:

‘We went from you know each area having their own Instagram page for both school nursing and health visiting and then Facebook pages for health visiting and school nursing…so we brought forward ChatHealth for both school nursing and health visiting. So yeah overnight our social media platforms took off …we haven't reduced our social media platform use (since returning to normal practice), you know that it's still a really big method for promotion and information sharing’ (FG/P16).

**Drawbacks—**Some SNs preferred having a physical presence in school and the personal interaction that in‐person education offered, noting that pre‐recorded videos could not substitute for this: ‘We were not always in school, so it was difficult to establish day to day concerns’ (Q/P29).

### 5.5 Developing the Infographic

Findings on the modes of practice identified and the considerations of their use were then integrated and presented in an evidence‐based infographic. Feedback was sought from the professional organisations in the original studies' consultation group, and delegates attending two professional conferences. The resource was amended accordingly ie adding a short section regarding context, and Quick Response code linking to further information about the original studies. The resource succinctly sets out the experiential evidence to raise awareness of the practice modes identified and encourages reflection on the usefulness of such changes in current practice and commissioning. The infographic was developed with the SN community in mind—as a quick reference, visually appealing resource and is in essence the findings of the study in an accessible format to inform SN practice (see Figure 1).

## 6 Discussion

The COVID‐19 restrictions led to SNs embracing a range of new and accelerated modes of practice to facilitate contact with CYP, offer health promotion advice and support, carry out assessments, and deliver care. This demonstrated the adaptability and resourcefulness of SNs as they strove to continue service delivery during these challenging times. A key finding was the wide range of practice modes employed and the varied benefits and challenges of each. There was no ‘one size fits all’ approach and the methods used were chosen or adopted out of necessity or availability of resources, and in response to the specific needs of individuals or groups. The two main changes in SN practice were the increase in digital modes of engaging with children and young people, and meeting with children and young people in places other than school.

SNs and young people alike appeared to appreciate the flexibility and convenience of video‐calling platforms and telephone contact. However, SNs also suggested that using virtual means could compromise their ability to build rapport with CYP, and many continued to prefer face‐to‐face contact as their primary method of engagement. Similar trends have been identified elsewhere; for example, a German study conducted during the pandemic found that in‐person contact was the preferred mode for mental health support among both practitioners and CYP (Stieger, Lewetz, and Willinger 2023). However, in the present study, SNs reported that CYP valued, and some even preferred, virtual contact methods such as video and telephone. These findings indicate that, in order to meet the needs of both SNs and CYP, there is a need for flexibility in the deployment of different methods to meet differing needs in practice. Virtual messaging such as WhatsApp or ChatHealth—where a young person can seek information or support anonymously, with a central number ensuring a response—was widely used by UK SNs in this study. This aligns with the findings of a content analysis of text transcripts from the ChatHealth platform used in a school health service for two inner London boroughs (England, UK) (Wales and Sayer 2019), where the text‐based platform facilitated a timely and efficient response to a range of physical, emotional, sexual health and appointment queries. The SNs interviewed for the Wales and Sayer (2019) study also noted how text platforms enhanced the visibility of the SN yet did not increase their workload.

However, SN participants across our own and other UK‐based studies (see Wales & Sayer 2015, Palmer 2023) have been clear that even high‐quality messaging services such as ChatHealth should not replace in‐person care, particularly for those with more serious and enduring difficulties. In‐person contact remains the ideal therapeutic modality of engagement, with new or expanded use of digital modes supplementing, rather than replacing, in‐person contact. A novel finding of this study was how virtual and text‐based platforms opened up a new avenue for CYP to access the SN service. Viewed through this lens, technology could be viewed as a gateway to in‐person contact with CYP by the SN. Nevertheless, it is important that such means of contact with the SN is one of several methods which includes in‐person contact, as not all CYP have equal access to technology (O'Donnell et al. 2022).

Throughout the pandemic, and as some restrictions were relaxed, different spaces for in‐person contact opened up, such as walk‐and‐talks and home visits. Walk‐and‐talks as an approach are growing in popularity and have an emerging evidence base. For example, therapy with an outdoor walk and talk element showed significant improvements in burnout, stress symptoms and general mental health and well‐being in a sample of adults (van den Berg and Beute 2021). Further, the benefit of the outdoors and nature is evidenced in many studies with CYP, particularly in relation to mental health therapy (see, for example, a metasynthesis of talking therapy in outdoor spaces by Cooley et al. 2020). However, there is a need for studies to specifically explore the acceptability and efficacy of walks‐and‐talks as a method of engagement, assessment and therapy with CYP, as this method was reported by SNs to have received a mixed reception from CYP themselves. Some SNs undertook home visits during pandemic restrictions. Whilst being a vital means for seeing and meeting with children who were unable to access school premises, they were also noted to offer the added benefit of being able to view a child's home context and observe family relationships and interactions. Whilst this is routine practice in health visiting, it is less common in school nursing. The benefits of home visiting are established in the literature on families with younger children. For example, the Family Nurse Partnership model, which supports young mothers from pre‐birth until their child is 2 years old, is based on a prescribed series of home visits with specific content to be covered within each visit (Olds and Yost 2020). Many young people are struggling with returning to school post‐pandemic (McDonald, Lester, and Michelson 2023). Therefore, this study indicates that home visiting could be an important means within school nursing to facilitate ongoing contact with CYP, and ensure their health needs are assessed and responded to. However, with reduced SN numbers nationally, whilst personalised and effective, the time required for a home visit is a challenge in a constrained service.

Specific challenges to CYP's health and well‐being continue in the wake of pandemic restrictions, such as increased mental health challenges (Owens et al. 2022; Moore et al. 2022), eating disorders (Gilsbach et al. 2022) and obesity rates (Anderson et al. 2023). There is a need for SNs to draw on the range of modes of practice identified during pandemic restrictions as these continue to be relevant as children experience ongoing health challenges. The infographic is a unique resource that has been developed through this study and is a first step in knowledge exchange based on experiential learning. The infographic has stand‐alone value as it has the potential to raise awareness of the range of different strategies that can be used to facilitate and/or enhance engagement with CYP and equip SNs with knowledge to foster innovative and responsive practice and critical reflection in a complex post‐pandemic landscape. The resource will also be used as the foundation for future work to develop a co‐created training resource for SN students (undertaking the SCPHN course) and/or continuing professional development (CPD) resource for established SNs.

Overall, this study has highlighted that there is no universal approach for SNs in supporting the health and well‐being of CYP. Different modes are useful for different situations, contexts and CYP's needs—for example, whether SN response takes a universal, personalised or specialist approach (SAPHNA 2021). However, the findings and resulting infographic are the first to bring together a range of tried‐and‐tested approaches and considerations for their use which has come from, and is informative for, the SN community of practice.

### 6.1 Strengths and Limitations

Data from 98 participants were included in the analysis and whilst rich, this is a small sample size which limits the generalisability of the findings. Commensurate with the aims of the study, data were gathered from UK‐based SNs and therefore findings may not be applicable to wider international contexts. However, the authors' international literature review of SN practice during the pandemic (Cook et al. 2022) suggests similar changes in practice modes internationally and knowledge and awareness of these different practices may also be of interest and relevance to SNs working in an international context. Data were captured at a particular point in time; some practices may or may not work as well in the post‐pandemic climate as they did during the pandemic. Focus groups were conducted online due to pandemic restrictions. Whilst this may have compromised group dynamics, this did not appear to be the case due to the lively discussion. Online focus groups also facilitated wider geographical reach and were more convenient for busy practitioners. Current findings represent SN perspectives only, we have not explored CYP and/or caregiver perspectives about the range of different modes. Some bias may be present through selecting specific elements of the datasets without consideration of the narrative context. The aim of the study was to examine more closely the responses to the questions asked in the survey and focus groups with regard to changes in mode of practice and considerations of these changes in practice through experience. As such, this study does not ask different questions of the data but hones in on one aspect within the data. As such, bias that could be introduced through asking different questions of the data has not been introduced. The visual and quick reference aspects of the infographic are strengths; however, it may be limited by the need to condense information and some nuance may be lost. The intention of the developed infographic in line with the study aim is to provide a summary of experiential learning and to be used as a prompt for reflection and discussion and consideration in a contemporaneous, localised context and is not designed to be an exhaustive generalisable resource.

## 7 Conclusion

Despite the aims of more widespread and systematic digitised healthcare, as set out in the NHS long‐term plan (2019), and the plethora of modes accelerated and adopted in school nursing as a result of pandemic restrictions, this has not yet been translated into clear recommendations or guidance for school nursing services. This is due in part to the rapidity with which changes were required during the pandemic, leading to wide variation in how modes (including technology) were used, a sense of trial and error, and little space for formal evaluation. There is a distinct lack of evidence for many of these modes, including those in use prior to the pandemic restrictions. For example, the National Institute of Clinical Excellence's guidelines on ChatHealth (2017), a popular platform used in school health, state that there is no comparative or qualitative evidence with relevant outcomes to demonstrate clinical effectiveness, and there has not been an update since 2017.

The authors' previous work (Cook et al. 2022; Sammut et al. 2022; Bekaert et al. 2023), together with the current analysis, is at the forefront of addressing this gap by drawing information together on the range of modes adopted to facilitate SN practice with CYP during the pandemic and considering potential post‐pandemic use. A formal evaluation of the effectiveness of the different modes is needed to establish clinical effectiveness. There is also a need for exploration of CYP's experiences and perspectives of the range of new and accelerated practices, and their usefulness in contemporaneous practice. A review to ascertain what is known about CYP and family members' experiences and understanding of SN support and complementary survey with CYP would add valuable insight into what CYP appreciate in relation to SN modes of practice.

This study aimed to identify and bring together information about different modes of practice and considerations for their use from the experiences of SNs in relation to practise during the COVID‐19 pandemic. In the next stage of developing a co‐created training resource for SCPHN students/CPD for SN the one‐page infographic can be used as an evidence‐based prompt for discussion. The one‐page infographic will be used to stimulate guided reflection (Johns 1984; Wilkinson 1999) on whether these modes of practice could be helpful in practice and what would need to be explored and set in place before employing these modalities, for example policy reviews and consent processes.

The infographic developed focuses on SN work with CYP; the data from the survey and focus‐group studies were also scrutinised for SN work in relation to the MDT and within SN teams. There is scope for the development of similar resources in these areas, and for developing further dimensions within the SCPHN training/SN CPD resource.

The unique and overarching picture from this study regarding SNs' work with CYP was the range of practice modes employed, and the varied benefits and challenges of each. There was no ‘one size fits all’ approach, and the modes used were chosen in response to the specific needs of individuals or groups. This study is one of the first to highlight that whilst extremely challenging, the pandemic restrictions were a springboard for innovation and there is ongoing opportunity for continued use of these new or expanded practices. The concise infographic is a novel quick reference guide to facilitate knowledge exchange within the SN community of practice and is grounded in experiential evidence garnered from a time when changes to practice were widespread and swift. This is a unique resource that increases awareness and stimulates discussion and reflection on the various practice modes available to SNs and their mode of delivery choices in current practice. Our next step, in partnership with the SN community, is to develop a package of activities around the resource that can be incorporated into SCPHN training, or as a CPD exercise for practising SNs.

## Author Contributions

All authors have agreed on the final version and meet at least one of the following criteria (recommended by the ICMJE*):

(1) substantial contributions to conception and design, acquisition of data, or analysis and interpretation of data;

(2) drafting the article or revising it critically for important intellectual content.

## Ethics Statement

This study received Oxford Brookes University Research Ethics Committee approval registration no. 211550. The original research was conducted as part of the project ‘Learning from the COVID‐19 pandemic in health and social care: Implications for nursing practice’, kindly funded by the General Nursing Council for England and Wales (GNCT) 2021 grant call.

## Conflicts of Interest

The authors declare no conflicts of interest.

### Peer Review

The peer review history for this article is available at https://www.webofscience.com/api/gateway/wos/peer‐review/10.1111/jan.16490.

## Data Availability

Data is stored in the university internal archive and is available on reasonable request.
